# Potential of Endangered Local Donkey Breeds in Meat and Milk Production

**DOI:** 10.3390/ani13132146

**Published:** 2023-06-29

**Authors:** Ante Ivanković, Gordan Šubara, Giovanni Bittante, Edmondo Šuran, Nicoló Amalfitano, Jasna Aladrović, Nikolina Kelava Ugarković, Lana Pađen, Mateja Pećina, Miljenko Konjačić

**Affiliations:** 1 Department of Animal Science and Technology, Faculty of Agriculture, University of Zagreb, Svetošimunska 25, 10000 Zagreb, Croatia; nkelava@agr.hr (N.K.U.); matejapecina@agr.hr (M.P.); mkonjacic@agr.hr (M.K.); 2 Agency for Rural Development of Istria, Ulica Tugomila Ujčića 1, 52000 Pazin, Croatia; gsubara@azrri.hr (G.Š.); edmondo.suran@azrri.hr (E.Š.); 3 Department of Agronomy, Food, Natural Resources, Animals and Environment (DAFNAE), University of Padova, Via dell’Università 16, 35020 Legnaro, Italy; bittante@unipd.it (G.B.); nicolo.amalfitano@unipd.it (N.A.); 4 Department of Physiology and Radiobiology, Faculty of Veterinary, Medicine University of Zagreb, Heinzelova 55, 10000 Zagreb, Croatia; jasna.aladrovic@vef.hr (J.A.); lana.padjen@vef.hr (L.P.)

**Keywords:** donkey, breeds, carcass and meat quality, milk quality, fatty acids

## Abstract

**Simple Summary:**

Animal genetic resources are a vital component of our genetic, economic, and cultural heritage, playing a crucial role in the economy, food production, regional identity, and ecosystem services. Local breeds, including donkey breeds that have lost their primary function, are often existentially endangered and require reintegration into alternative economic utilization programs. Donkey meat and particularly donkey milk have attracted consumer interest due to their nutritional and functional value. This research focused on the production and quality indicators of donkey meat and milk, aiming to establish and optimize sustainable economic utilization programs for donkeys. This approach represents the most effective strategy for long-term in situ conservation programs of endangered local donkey breeds in their natural habitats. The research outcomes provide a solid basis for the development of similar programs for other local donkey breeds.

**Abstract:**

The problem of the erosion of animal genetic resources is evident in certain local donkey breeds, and their long-term sustainability can be achieved by economically repositioning them. To develop alternative and sustainable commercial programs, the meat and milk production characteristics of Istrian donkey and Littoral Dinaric donkey breeds were investigated. The meat production characteristics were examined in mature males, whose carcasses were dissected, and meat composition was determined using NIT spectrophotometry and gas chromatography. Milk yield and milk composition were determined in jennies in second or subsequent lactations by measuring milk volume and using infrared spectrometry and gas chromatography. Compared to the Littoral Dinaric donkey, the Istrian donkey has a higher carcass weight and dressing percentage (*p* < 0.001). The share of boneless meat in relation to live weight was 28.27% in the Istrian donkey and 26.18% in the Littoral Dinaric donkey. The absolute masses of primal cuts of meat in E, I, and II classes were significantly greater in Istrian donkeys than in Littoral Dinaric donkeys (*p* < 0.01), although the differences in the proportions of primal cuts were not significant. The breed did not have a significant impact on the color, pH, or meat composition. A significant influence of breed on milk yield, lactose, protein, and the fat content of milk was observed (*p* < 0.01). A significant influence of breed on the ratio of n-6/n-3 PUFA fatty acids in donkey milk was observed (*p* = 0.002). The values of the atherogenic and thrombogenic indexes were favorable, considering potential beneficial effects of donkey milk and meat on consumer health. The findings of this research suggest that local donkey breeds hold significant potential for meat and milk production, focusing on the uniqueness and quality of their products rather than the quantity of meat and milk they can produce.

## 1. Introduction

The donkey was domesticated in Africa around 5000 B.C.E, followed by further expansions in this continent and Eurasia, and ultimately returning to Africa [1,2,3]. They were the hardiest transport animals in ancient Africa and the Near East, provided reliable access to meat and renewable resources such as milk and blood, and were used for labor for heavy tasks including plowing, turning grindstones, and pumping water from wells [4]. The development (modernization) of agricultural production and the introduction of modern means of transport in the 20th century led to a gradual loss of the primary function of donkeys (work, transport), which was especially pronounced in the more economically developed parts of the world. In some parts of the world, local donkey breeds have been marginalized to the point that their biological persistence is no longer assured, and some donkey breeds have become permanently lost (extinct). Considering the direct and indirect benefits of local donkey breeds, their genetic, economic, social, and cultural potential is now recognized as a valuable genetic resource essential for the sustainability and security of food production, identity preservation, rural vitality, and functionality, and the overall biodiversity of various ecosystems. According to the FAO report, 178 donkey breeds have been recorded worldwide, 90% of which are of local character. It is known that only 5.62% of donkey breeds are not at risk of extinction, while 24.72% of breeds have some risk of extinction [5]. For 68.5% of donkey breeds, there are no relevant indicators of biological endangerment.

Local donkey breeds in Croatia, such as the Istrian donkey and the Littoral Dinaric donkey, faced a severe threat to their biological viability at the end of the 20th century. Currently, the Istrian donkey population with offspring is approximately 860 animals, while the Littoral Dinaric donkey population is around 3680 animals [6]. The Istrian donkey and the Littoral Dinaric donkey have been described in terms of conformation [7] and genetic structure [8]. However, ensuring the long-term sustainability of these local donkey breeds requires their economic repositioning, the creation and optimization of economic utilization programs, and improved collaboration among all stakeholders. While the revitalization of the working function of donkeys is not anticipated, there is a growing opportunity for the economic reaffirmation of local donkey breeds through the production and marketing of donkey milk and meat. The commercialization of these products has not flourished due to product scarcity, low production rates, and consumer preferences [9]. However, encouraging the production of donkey meat and milk serves multiple purposes: preserving endangered local donkey breeds, obtaining a healthy product, and conserving the natural resources that offer environmental and social benefits to rural areas.

Donkeys primarily serve as working animals and are not commonly used for meat production, which has resulted in a limited number of scientific studies on the topic. The top three countries in terms of donkey meat production are China, Niger, and Burkina Faso [10]. However, there is also a tradition of consuming donkey meat in certain European countries, such as Italy, and specific regions of certain countries, such Istria in Croatia. Researchers have conducted studies on the slaughter characteristics and meat quality of certain donkey breeds. Polidori et al. [11] observed a dressing percentage of 53.3% in male donkeys of the Martina Franca breed. They also found that the age of the donkeys influenced the composition and tenderness of the meat [12]. The rearing system had an impact on average daily gain, carcass weight, and certain meat quality parameters [13]. Pinto et al. [14] noted that the use of ω-3-enriched feed led to differences in the fat, meat, and bone content of the shoulder and loin, as well as the ash content of the meat. Donkey meat has been found to have a high protein content (22.8%), low levels of lipids and cholesterol (2.02%; 68.7 mg/100 g) [11], and a beneficial fatty acid composition [15]. The meat contains a high concentration of polyunsaturated fatty acids, a favorable ratio of unsaturated fatty acids (UFA) to saturated fatty acids (SFA), and a substantial amount of essential amino acids, thus confirming its favorable nutritional value compared to other types of red meat [16].

Compared to research on donkey meat, there has been a larger number of studies focused on the milk yield and chemical composition of donkey milk. The variability of milk yield in different donkey breeds is large. For example, Ragusana and Martina Franca jennies have a milk yield ranging from 0.59 to 1.08 kg [17,18,19,20,21]. Jiangyue jennies have produced 1.0 kg of milk [22], while Littoral Dinaric jennies, under extensive feeding conditions, yielded only 172 mL per milking [23]. Total fat, protein, and mineral content were influenced by the stage of lactation [24]. In the milk of different donkey breeds, the lactose content ranged from 5.75% to 7.32% [25,26], the protein content from 1.42 to 2.04% [17,20], the milk fat content from 0.11% to 1.16% [20,22], and ash content from 0.35% to 0.67% [26,27]. The somatic cell count (SCC) in the milk of Ragusana, Amiata, Littoral Dinaric, and Cypriot jennies was below 4.4 log10 mL^−1^ [17,18,23,24,28]. Although the microbiological quality of donkey milk is generally good [23,24,25,29], potential pathogenic microbe species were identified in donkey milk, emphasizing the need for quality control measures to be taken for health and safety prior to human consumption [29].

In order to achieve the long-term and sustainable conservation of local donkey breeds as valuable animal genetic resources, it is crucial to combine efficient and balanced economic utilization with conservation measures. Therefore, the objective of this research is to assess the variations in meat and milk yields and compositions between the Istrian donkey and Littoral Dinaric donkey breeds within traditional farming systems.

## 2. Materials and Methods

### 2.1. Animals, Farming Systems, and Experimental Design

All animals used in this study originated from traditional farms in the geographical area of Dalmatia (Littoral Dinaric donkey) and Istria (Istrian donkey). During their life, the animals were kept in traditional housing conditions, free-range and with unhindered seasonal access to pasture. The feeding of the donkeys was based on grazing on modest Mediterranean and sub-Mediterranean pastures and hay, supplemented with concentrates (grains) in amounts of about 1.1% of BW (Body Weight). Since the aim of the research was to determine the meat and milk production and quality of donkeys raised in local traditional conditions, the animals were not exposed to any intensive feeding regime.

The study of slaughter characteristics of the two local donkey breeds was conducted over two years (2020–2022) on a total sample of 60 males aged three to ten years. Of the animals included in the research, thirty belonged to the Istrian donkey breed and thirty to the Littoral Dinaric donkey breed. Istrian donkeys were collected from eight donkey farms in Istria and the Littoral Dinaric donkeys were collected from three farms in Dalmatia.

The study on milking characteristics of two local donkey breeds was conducted over a two-year period (2020–2022) on a total sample of 80 jennies, of which 40 were Istrian jennies and 40 were Littoral Dinaric jennies. Istrian jennies were located on three farms in Istria (Pula, Pazin, Rovinj) and Littoral Dinaric jannies were located on two farms in Dalmatia (Zadar, Imotski). All jennies included in the study were older than five years and were in the second lactation or later. The feeding of the lactating jennies was based on hay and pasture, supplemented with concentrates (grains) in amounts from 1.0 to 1.5 kg/day for the Littoral Dinaric jennies, and 1.5 to 2.0 kg/day for the Istrian jennies.

### 2.2. Carcass and Meat Characteristics

The donkeys were transported by truck from the farm to the slaughterhouse immediately before the slaughter (the animals were not rested in the livestock depot). Before transport, the animals were fasted for 18 h, with drinking water available. The transportation time for the animals was kept within a maximum interval of 2.5 h. Immediately before slaughter, the body weight of the animals was weighed.

The donkeys were slaughtered immediately after unloading. Slaughter was performed in a slaughterhouse approved by the European Union to slaughter ungulates according to a standard procedure respecting the norms of European Union that included stunning with a compressed air gun with a penetrating wedge, cutting the large jugular veins (*vena jungularis*) in the hanging position for bleeding, skinning, the removal of abdominal and thoracic cavity organs, final cleaning and processing of the carcasses, and the placement of the carcasses in the cooling chamber. Carcasses were chilled under commercial conditions for 24 h at 4 °C.

The cooled donkey carcasses were weighed the next day. They were moved to the cold room for cutting (4 °C). Samples of the Longissimus Thoracis et Lumborum (LTL) with bone were taken from the left side of each carcass between the 10th and 18th ribs to measure the pH_24_, color, and meat composition and to assess the ratio of muscle, bone, fat, and connective tissue. Then, the remaining part of the left and the right half of the carcass were cut according to the gastronomic cuts (loin, tenderloin, neck, big rose, small rose, black fricandeau, knuckle, ribs and flank, shoulder, shank, other cuts/rest of meat; Supplementary Figure S1), and the bones were finally separated from the meat (deboning).

pH value was measured 24 h post mortem (pH_24_) with a Eutech CyberScan pH 310 instrument at the site where the LTL was located, between the 10th and 11th thoracic vertebrae. On the same section (between the 10th and 11th thoracic vertebrae), the surface of the LTL was drawn on a transparent foil and then measured with a Robotron planimeter (Reiss Precision, Germany). The color of the meat was measured on the cross-sectional area of the LTL (between the 10th and 11th thoracic vertebrae). Color was measured after bloom time (80 min after cutting, with the LTL exposed to air). To evaluate the color pattern, the CIE (Comission Internationale de l’Eclairage) values (L* (Lightness), a* (Redness), and b* (Yellowness)) were measured using a Minolta Chroma Meter CR-410 colorimeter (Minolta Co., Ltd., Tokyo, Japan) with a measuring surface of 50 mm diameter and a D65 illumination.

A sample of the rib section comprising the 11th to 18th thoracic vertebrae was collected, and its dissection was used to estimate the tissue content of the carcass (muscle/bone/fat/connective tissue). A chemical analysis of the meat was performed on a meat sample of MLT from the 11th to 12th rib (weight 100 g) using NIT spectrophotometry (near-infrared transmission spectroscopy) in the measuring range 850–1050 nm with a food-scan instrument (Foss Electric A/S, Hillerød, Denmark).

The analysis of fatty acid content in total meat lipids was performed on 20 meat samples, 10 samples from each of two local donkey breeds selected at random. LTL meat samples for fatty acid analysis were collected from the 13th to 14th rib (weight 100 g), vacuumed after collection, and stored at −20 °C until analysis.

### 2.3. Milking and Milk Analysis

The amount of milk was estimated in the period between 60 and 100 days of lactation. The milking of the donkey mares was carried out with a milking machine, once in the morning (at 9:00 a.m.). The milking machine operated with a vacuum level of 42 kPa, pulse rate of 120 cycles/min, and pulse ratio of 50%. Before the actual milking, offspring were separated from the jennies for three hours in the neighboring pen, maintaining visual contact.

After milking, the amount of milk was measured with a measuring cup and placed in the refrigerator. The pH was measured in the cooled milk using an Eutech CyberScan pH 310. The milk samples (200 mL) were stored in bottles with preservatives at 4 °C to analyze the milk composition, somatic cell count, and microorganisms in the milk. The percentage of dry matter, milk protein, fat, and ash are determined by the method of infrared spectrometry with MilkoScan FT 6000. The number of somatic cells (SSC) was determined using the fluoro-opto-electronic method using a Fossomatic 90. The total number of bacteria (MC) was determined by the flow cytometry method using the Bactoscan FC.

The analysis of the content of fatty acids in the total lipids extract of donkey milk was performed on 32 milk samples, 16 milk samples from each of the two local breeds selected at random. Milk samples for fatty acid analysis in a volume of 120 mL were cooled immediately after milking and then frozen at a temperature of −20 °C and stored in this way until analysis.

### 2.4. Analysis of the Composition of Fatty Acids in Lipids of Donkey Meat and Milk

The extraction of total lipids from donkey meat and milk was performed using a modified method according to Folch et al. [30]. Donkey milk was thawed, and then the extraction of total lipids was performed using a chloroform/methanol/water solvent mixtures with the final ratio 2:2:1.8. After thawing, samples of donkey meat were homogenized in 0.14 mol/L KCl for 60 s at 13,500 rpm with an Ultra-Turrax T25 Basic homogenizer (IKA, Staufen, Germany). The extraction of total lipids from donkey meat was performed using a solvent extraction mixture of chloroform/methanol, which was divided into three parts according to their concentration ratios: 2:1, 1:1, and 1:2. Total lipid homogenates were extracted for 30 min with stirring (700 rpm) and then centrifuged for 10 min at 3000 rpm at 20 °C. Total lipid extracts from milk and meat were concentrated in a UNIVAPO 100H evaporator, equipped with a UNICRYO MC 2L cooling unit (Uniequip, Planegg, Germany). Fatty acids from the total lipid extract were converted to methyl esters via trans-esterification according to ISO 14156-IDF 172: 2001 and ISO 15884-IDF 182: 2002 (milk samples), and ISO 5509: 2000 (meat samples). The analysis of fatty acid methyl esters was performed on a gas chromatograph (Agilent 8860; Agilent Technologies Inc., Santa Clara, CA, USA) equipped with a flame ionization detector (FID). Temperatures of the injector and detector were 200 °C and 240 °C, respectively. Chromatography was performed on a DB-23 capillary column (Agilent Technologies, Santa Clara, CA, USA). The initial column temperature was 120 °C for 3 min, then it was increased to 260 °C by heating for 6 °C/min and was held at that temperature for 5 min. Hydrogen was used as carrier gas at a flow rate of 1 mL/minute. The collection and processing of the results were carried out using the computer program OpenLAB CDS ChemStation Workstation VL. Fatty acids were identified by a comparison of the retention times with methyl standards (Sigma Aldrich Chemie, GmbH and Supelco, St. Louis, MO, USA). Quantification was carried out using the methyl nonadecanoic acid (C19:0). Fatty acid composition was calculated as the percentage of each individual fatty acid relative to the total fatty acids.

The atherogenic index (AI) and thrombogenic index (TI) were calculated according to Ulbricht and Southgate [31] and were calculated using the equations AI = (C12:0 + 4 × C14:0 + C16:0)/(MUFA + PUFA) and TI = (C14:0 + C16:0 + C18:0)/[(0.5 × MUFA) + (0.5 × PUFA n-6) + (3 × PUFA n-3) + (PUFA n-3/PUFA n-6)]. Stearoyl-CoA desaturase (SCD) activity indices were estimated by computing the ratio of product/(substrate + product) in meat and milk: SCDi-16 = [C16:1/(C16:1 + C16:0)] × 100; SCDi-18 = [C18:1/(C18:1 + C18:0)] × 100.

### 2.5. Statistical Analysis

Results were processed using the general linear model (GLM) procedure using Statistical Analysis Software (SAS) (SAS Inc., Cary, NC, USA). The effects of donkey breeds on phenotype (carcass characteristics, meat and milk composition) were determined using the model:

Y_ij_ = μ + α_i_ + β_ij_+ ε_ij_

where Y_ij_ is the value of the analyzed trait, μ is the overall mean, α_i_ is the effect of donkey breeds (ID, LDD), and ε_ij_ is a random error. The age of donkeys and days of lactation were included as covariables (β_ij_) in the model. The differences between means were estimated using Tukey’s test. All tables contained the least square mean (LS Mean) and standard error (SE) of the means. Pearson correlation coefficients were calculated with IBM SPSS statistics software, version 21 (Armonk, NY, USA).

## 3. Results

### 3.1. Donkey Meat

The slaughter and dissection traits of two local donkey breeds are presented in Table 1. The average age of Istrian donkeys at slaughter was 6.43 ± 0.46 years, while the Littoral Dinaric donkeys were slightly younger (5.83 ± 0.36 years), on average. The average slaughter weight of Istrian donkeys was significantly higher (+54%) than that of Littoral Dinaric donkeys, reflecting the size difference between the two breeds. Obviously, the processed carcasses of Istrian donkeys also had a significantly higher weight (+56%) compared to those of Littoral Dinaric donkeys, and the dressing percentage was slightly but significantly larger (+0.73%).

The dissection of the rib section did not reveal any significant differences in the proportion of muscles and bones between the Istrian donkey and the Littoral Dinaric donkey (Table 1). However, there was a lower proportion of fatty tissues (−11%) in the Istrian donkey compared to the Littoral Dinaric donkey. The LTL (Longissimus thoracis et lumborum) surface area of the Istrian donkey was significantly larger (+16%) than the LTL surface area of the Littoral Dinaric donkey, and this difference can be largely explained by the difference in carcass size. The LTL surface area was positively correlated with the cold carcass weight (0.745; *p* < 0.001), dressing percentage (0.411; *p* = 0.01), and proportion of muscle in the rib section (0.519; *p* = 0.007). As expected, the proportion of muscle in the rib section was negatively correlated with the proportions of bone (−0.843; *p* < 0.001) and fat tissue (−0.597; *p* = 0.001).

The share of deboned meat in carcasses suitable for gastronomic purposes was 56.27% (50.10 kg) for the Istrian donkey and 52.86% (30.13 kg) for the Littoral Dinaric donkey. The dissection of the carcasses into primal cuts revealed significant differences in the absolute weight of cuts between the Istrian donkey and the Littoral Dinaric donkey (*p* < 0.01). However, there were no significant differences in the share (%) of primal cuts from boneless meat, except for the proportion of boneless neck (13.26% vs. 10.82%; *p* < 0.001). The yield of deboned primal cuts suitable for gastronomic purposes obtained from carcass jointing was significantly greater (+66%) in Istrian donkeys than in Littoral Dinaric donkeys, when expressed in kg/animal. Moreover, the differences were significant when expressed as a proportion of the cold carcass weight (Table 1).

As expected, the dissection of carcasses into primal cuts revealed significant differences in the absolute weight of all cuts in favor of the Istrian donkeys (+39% for fillet, to +105% for neck). However, when the meat cuts were expressed as a percentage of total boneless meat, the differences between the two breeds were not significant, except for the proportion of boneless neck, which showed a 23% difference. The proportion of extra class cuts was slightly higher in the Istrian donkey compared to the Littoral Dinaric donkey (28.32 vs. 27.51%). The proportion of first-class primal cuts in the total deboned meat was lower in the Istrian donkey than in the Littoral Dinaric donkey (30.80 vs. 30.58%). Similarly, the share of primal cuts of second-class meat cuts was slightly lower in the Istrian donkey than in the Littoral Dinaric donkey (41.10 vs. 41.69%). In terms of absolute values, the mass of meat cuts in classes E, I, and II was higher in the Istrian donkey (14.19, 15.32 and 20.59 kg) than in the Littoral Dinaric donkey (8.29, 9.28 and 12.56 kg) (Table 2).

The physical and chemical characteristics of Istrian donkey and Littoral Dinaric donkey meat are presented in Table 3. The pH_24_ values of chilled Istrian donkey and Littoral Dinaric donkey meat were favorable (<5.70), indicating an appropriate slaughter process without unnecessary stress. The color values of the meat were acceptable, especially for Istrian donkey carcasses (L* = 33.15, a* = 10.48, b* = 12.22; C* = 16.17; H* = 49.43). A significant correlation was observed between the pH_24_ values and the L* value of the meat color (−0.677; *p* = 0.006). The difference in moisture content between Istrian donkey and Littoral Dinaric donkey meat was not significant (72.63%, 72.10%), and neither were the differences in protein content (23.56%, 23.63%), fat content (1.77%, 2.10%), or ash content (1.13%, 1.16%). The fat content in donkey meat negatively correlated with moisture content (−0.575; *p* < 0.001) and protein content (−0.571; *p* < 0.001). In terms of the relationships between the composition and meat color, a correlation was found between lightness (L*) and protein content (0.405; *p* = 0.003), fat content (−0.482; *p* < 0.001), and moisture content in donkey meat (0.286; *p* = 0.040). Additionally, a significant correlation was observed between redness (a*) and protein content (−0.511; *p* < 0.001), as well as fat content in the meat (0.447; *p* = 0.001).

Unsaturated fatty acids dominated in the meat of both Istrian donkey and Littoral Dinaric donkey, accounting for 54.89% and 54.62%, respectively, with oleic fatty acid (C18:1c-9) being the most prevalent (Table 4). Among the saturated fatty acids, palmitic acid (C16:0) was the dominant one in the intramuscular fat of both Istrian donkey and Littoral Dinaric donkey meat, comprising 23.75% and 24.03%, respectively. Polyunsaturated fatty acids (PUFA) were mainly represented by linoleic fatty acids (C18:2n-6), constituting 17.19% and 18.07% of the intramuscular fat in Istrian donkey and Littoral Dinaric donkey meat, respectively. However, the differences in the proportions of individual fatty acids and total fatty acid content in the intramuscular fat of Istrian donkey and Littoral Dinaric donkey were not significant.

The n-6/n-3 PUFA index values in the intramuscular fat of donkey meat are favorable for consumers, with values of 2.79 and 3.17 achieved for the two Croatian donkey breeds, respectively. This is particularly noteworthy considering the beneficial effects on human health. The indices values of AI (0.66, 0.70), TI (0.82, 0.86), SCDi 16 (14.78, 14.79), and SCDi 18 (71.85, 71.80) did not show any significant differences between the Croatian local donkey breeds.

### 3.2. Donkey Milk

The milk yield, pH, chemical composition, and microbiological quality of milk from Istrian jennies and Littoral Dinaric jennies are presented in Table 5. Milk yield was significantly higher in Istrian jennies compared to the milk yield of Littoral Dinaric jennies (+399.5 mL/milking; *p* < 0.001). The raw milk pH was also higher in the milk of Istrian jennies than in milk of Littoral Dinaric jennies (+0.19; *p* < 0.001). Regarding the milk composition, the lactose content was significantly higher in the milk of Istrian jennies compared to Littoral Dinaric jennies (+0.3 g/100 g; *p* < 0.001). On the other hand, the milk of Littoral Dinaric jennies had a higher protein content (+0.24 g/100 g; *p* = 0.001) and fat content (+0.22 g/100 g; *p* < 0.001). However, there were no significant differences observed in the number of somatic cells and the number of microorganisms in the milk between the two Croatian local donkey breeds.

Furthermore, milk yield showed a positive correlation with milk fat content (0.408; *p* < 0.001) and a negative correlation with milk protein content (−0.277; *p* = 0.013). The lactose content in milk was negatively correlated with milk fat content (−0.459; *p* < 0.001) and milk protein content (−0.546; *p* < 0.001).

In the milk of both local donkey breeds, the fatty acids C14:0, C16:0, C18:1n-9, and C18:2n-6 were found to be present in concentrations higher than 10 g/100 g. Significant differences were observed between the milk of Istrian jennies and Littoral Dinaric jennies in terms of the content of C9:0, C20:1, and C18:3n-3 fatty acids, (Table 6).

No significant breed effect was found on the share of saturated fatty acids (SFA), monounsaturated fatty acids (MUFA), and polyunsaturated fatty acids (PUFA). However, the milk of Istrian jennies had a higher content of n-3 PUFA compared to the milk of Littoral Dinaric jennies (+2.43 g/100 g of total fatty acids; *p* = 0.014), resulting in a more favorable n-6/n-3 PUFA ratio in the milk of Istrian jennies (2.35 vs. 4.97; *p* = 0.002). The influence of donkey breeds on the values of SCDi 16 and SCDi 18 indices was not significant. The atherogenic index (AI) did not differ between donkey breeds, while the thrombogenic index (TI) was significantly higher in Littoral Dinaric jennies’ milk (+0.30; *p* = 0.03).

## 4. Discussion

### 4.1. Donkey Meat

The live weight of Istrian donkeys is significantly higher than the live weight of Littoral Dinaric donkeys (+62.1 kg), which was also reflected in the greater mass of the cold carcass (+32.04 kg; *p* < 0.001). The larger body frame of the Istrian donkey compared to the Littoral Dinaric donkey was also observed in an earlier study, with a difference of +24.17 cm in wither height. This difference can be attributed to their specific genetic constitution and the agroecological conditions of their rearing [7]. Interestingly, local breeds in the Istrian peninsula area, such as the Istrian donkey, Istrian cattle, Istrian goat, and Istrian sheep, have a larger body frame and body mass compared to other local breeds of the same species in Croatia. This can be attributed to the unique environmental factors and centuries-old specific breeding strategies employed in the region. The Istrian donkey, due to its size, belongs to a group of medium to large donkey breeds, similar to the Italian breeds Ragusano, Pantelleria, Romagnolo, or Martina Franca. The Martina Franca breed was utilized in the selection process in Istria during the early 20th century to achieve the desired physical characteristics of the donkeys [32]. On the other hand, the Littoral Dinaric donkey belongs to a group of smaller donkey breeds, with an average wither height of about 100 cm, similar to other donkey breeds commonly found in Southeastern Europe. The dressing percentage was higher in the Istrian donkey compared to the Littoral Dinaric donkey, with values of 50.25% and 49.52%, respectively. However, this was lower than the dressing percentage observed in donkeys from the Botswana region [33] or the Martina Franca donkeys, which recorded a dressing percentage of 53.3% [11]. In a study conducted by Polifori et al. [13], crossbred male animals (Martina Franca × Ragusana; age 16 months) raised under extensive management conditions showed a dressing percentage of 51.3%. It is worth noting that the animals included in the present study did not receive additional concentrate and were older. Therefore, it can be concluded that the dressing percentage of the studied donkey breeds was relatively favorable and had the potential for improvement through selection or the implementation of an intensive feeding system [13].

In the valorization and economic utilization of local donkey breeds, particularly in the context of slaughterhouses and restaurants offering gastronomic specialties, the percentage of deboned meat in the carcass serves as a crucial indicator. This percentage determines the purchase price of live donkeys, the price of the donkey carcass, and the sale price of meat categorized by primal cuts. The determined ratios between the weight of live animals and the weight of the carcass and boneless meat of the Istrian donkey (177.20:89.04:50.10 kg) and the Littoral Dinaric donkey (115.10:57.00:30.13 kg) served as references for establishing and optimizing the average market prices of “live donkeys/carcass/boneless meat”. It was observed that the proportion of boneless meat in relation to the live weight of donkeys was higher in the Istrian donkey compared to the Littoral Dinaric donkey (28.27% vs. 26.18%). The aforementioned findings regarding carcass usability indicated that it was justifiable to offer a higher price for Istrian donkeys, as the proportion of boneless meat in relation to live weight was approximately 2% higher compared to Littoral Dinaric donkeys. However, it is worth noting that the percentage of muscle tissue in the “rib section” (66.05–67.10%) was higher than the overall meat content in the whole carcass determined by the complete deboning of the entire carcass (54.39%, 55.64%). As a result, it can be challenging to accurately estimate the muscle/bone/fat and connective tissue ratio. For future studies, it would be necessary to precisely determine the anatomical position of the vertebral column in order to assess the proportions of meat, bone, fat and connective tissue in the donkey carcass. The values of “muscle/bone/fat and connective tissue” determined through the dissection of the “rib section” should not differ significantly from the values obtained through the dissection (deboning) of the entire carcass.

The pH_24_ values of Istrian donkey and Littoral Dinaric donkey meat fell within the favorable range recommended by Wulf and Wise [34], which suggests that the pH_24_ of meat should ideally range from 5.3 to 5.7. The observed pH_24_ values of 5.50 and 5.56 in the meat of Istrian donkeys and Littoral Dinaric donkeys indicated favorable degradation processes of glycogen that remains in the muscle after slaughter. A favorable pH_24_ also indicates that there was no significant stress experienced by the animals before slaughter, which is beneficial for the meat’s sustainability and its suitability for processing. In a study by Polidori et al. [11], a pH_24_ value of 5.57 was determined in the meat of the Martina Franca donkey breed. The parameters of meat color did not show significant differences between the Istrian donkey and Littoral Dinaric donkey meat. Applying the recommendations for meat categorization according to Wulf and Wise [34], based on the L* value, the meat could be classified as dark meat (category 1; L* < 33.5). However, considering the b* value, it could be categorized as having favorable meat colors (category 8; b* > 12.00). In a study by Polidori et al. [13], color parameters (L*, a*, b*) were observed in young male animals (16 months old) raised under extensive rearing conditions, resulting in values of 34.4, 12.1, and 7.6, respectively. Animals reared intensively exhibited slightly different color parameters, with values of 35.7, 11.4, and 7.7 for L*, a*, and b*, respectively. Regarding the aforementioned research [13], the lower values of the L* color parameter and higher values of the b* color parameter observed in the meat of Istrian donkeys and Littoral Dinaric donkeys could be primarily attributed to their older slaughter age and the extensive grazing rearing method.

**Figure 1:**
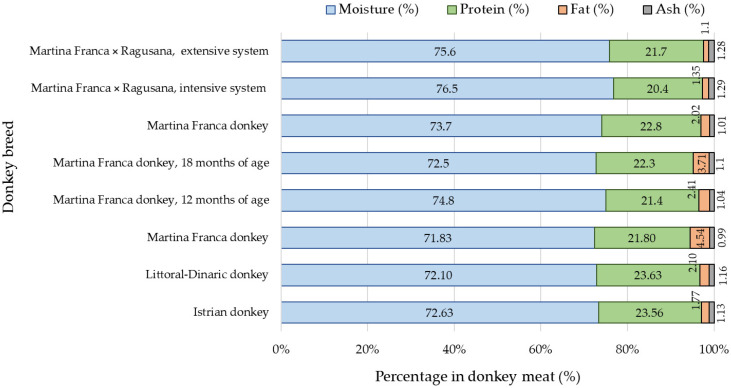
Average values of moisture, protein, fat, and ash content in meat of different donkey breeds (according to different authors) [11,12,13,14].

Regarding the composition of the meat of the Istrian donkey and the Littoral Dinaric donkey, no significant differences were found in terms of moisture, protein, fat, and ash content. Pinto et al. [14] confirmed the lower moisture, protein, and ash content, as well as the higher fat content in the meat of 12-month-old Martina Franca donkeys, which could be partially explained by their age and being fed with a higher energy meal (Figure 1). In the meat of 12-month-old donkeys of the same breed, a higher moisture content and lower protein and fat content were determined [13]. At 15 months of age, the meat showed lower contents of moisture, fat, and ash, but higher protein content [11]. Additionally, the protein and fat contents in donkey meat were lower at 18 months of age [12]. Under extensive management conditions, Polidori et al. [13] found higher moisture and ash content, as well as lower protein and intramuscular fat content, in the meat of male crosses (Martina Franca × Ragusana). The observed variations in the meat composition can be explained by factors such as age, sex, breed, and management practices (including feeding), as supported by previous studies [11,13].

Regarding the individual fatty acids, four fatty acids dominated, including two saturated (C16:0, C18:0) and two unsaturated (C18:1n-9, C18:2n-6), which aligns with findings from previous studies [13,15]. When considering the fatty acids composition in the intramuscular adipose tissue of the Istrian donkey and Littoral Dinaric donkey, UFA dominated, with a higher proportion of MUFA compared to PUFA. Previous studies [13,14,15] also reported a lower proportion of SFA compared to UFA (41.48% vs. 58.52%; 38.51 vs. 61.49%). The content of PUFA in the intramuscular fat tissue of Istrian donkey and Littoral Dinaric donkey meat (24.21%, 24.59%) was higher than the determined content in the Martina Franca donkey meat (13.02, 16.55%) [14], but lower than the share of 28.08% in extensively reared Martina Franca × Ragusana crosses [13]. The determined values of the atherogenic index (0.66, 0.70) were higher than those reported by Polidori et al. [13], but lower than the values determined by Polidori et al. [11]. The content of n-3 PUFA in the intramuscular adipose tissue of Istrian donkey and Littoral Dinaric donkey meat was higher than the values determined in previous studies [13,15]. From the nutritional and health perspectives, the overall indicators of meat composition and the fatty acids profile in the intramuscular adipose tissue of donkey meat indicated that it was a favorable food choice.

The identified indicators of carcass and meat characteristics, such as pH, color, and chemical composition, play a crucial role in optimizing the production program and marketing of meat from local donkey breeds. These indicators help to ensure that the meat meets the preferences and expectations of consumers in regions where donkey meat is traditionally consumed. For instance, in the Istrian peninsula, which is the breeding area of the Istrian donkey, donkey meat has a long-standing tradition of consumption. On the other hand, in the breeding area of the Littoral Dinaric donkey, donkey meat consumption is not common. Modern consumers have shown a growing interest in leaner meat with low fat content, and also pay great attention to animal feeding management [35]. Therefore, by considering these consumer preferences and aligning production practices accordingly, the marketability and acceptance of donkey meat can be enhanced. This includes ensuring appropriate feeding strategies to achieve desired meat characteristics and meeting the demand for lean and high-quality meat products. By responding to consumer expectations and preferences, local donkey breeds can effectively cater to the evolving market demands and promote their meat in a sustainable and consumer-friendly manner. The established quality indicators of local donkey breeds serve as valuable tools to educate existing consumers and attract new consumers to donkey meat. The objective is to promote the increased consumption of donkey meat, thereby boosting the demand for this type of meat. This increased demand not only contributes to the reproductive efficiency and self-sustainability of local breed populations, but also supports the overall viability of these breeds. To effectively promote donkey meat, it is important to utilize meat productivity indicators in determining the pricing of live animals intended for meat production, as well as the final meat products, taking into account the level of finishing. By incorporating these indicators into the pricing structure, the value and market positioning of donkey meat can be accurately reflected. By focusing on enhancing consumer awareness and understanding of the quality attributes of donkey meat, coupled with appropriate pricing strategies, the goal of increasing consumption and creating a sustainable market for donkey meat can be achieved. This, in turn, will contribute to the long-term preservation and viability of local donkey breed populations. Considering the ratio of live animal weight, carcass weight, and boneless meat for Istrian donkeys (SBW:CCW:BMW) as 100.00:50.25:28.27, the minimum price per kilogram of live weight, carcass and meat (excluding transport, slaughter, cutting, and other costs) would be 1.00:1.99:3.54. For Littoral Dinaric donkeys, using the same indicators (100.00:49.52:26.18), the minimum price ratio would be 1.00:2.02:3.82 for a kilogram of live weight, carcass, and meat (excluding additional costs). To illustrate with the example of Istrian donkeys, if the price of the live donkeys was set at 4.00 EUR/kg, the minimum price ratio for live weight, carcass, and meat would be 4.00:8.09:15.28 EUR/per kg. These production and quality indicators of local donkey breeds in meat production serve as valuable insights into the donkey breed’s potential, and can be utilized in a well-balanced program for its economic exploitation.

### 4.2. Donkey Milk

The significantly higher milk yield of Istrian donkeys compared to Littoral Dinaric donkeys can be primarily explained by the larger body size of Istrian donkeys [7]. In a previous study [23], Littoral Dinaric donkeys raised under extensive feeding conditions (hay and pasture without concentrate) yielded 180.7 mL/milking in the third month of lactation. However, in the current study, Littoral Dinaric donkeys fed a daily mixture of oats and barley ranging from 1.0 to 1.5 kg/day achieved a milk yield of 443.1 mL/milking. Salimei et al. [21] reported a milk yield of 740 mL/milking for the Martina Franca and Ragusana breeds. Other studies have shown that the milk yield of Ragusana jennies ranged from 590 mL/milking to 1080 mL/milking [17,18,19,20,21], and that of Amiatia jennies ranged from 379.08 mL/milking [27] to 771.11 mL/milking [28]. Various factors influence milk yield. Research has shown that extending the interval between milkings to 8 h instead of 3 h and conducting milking in the morning rather than in the evening results in a higher milk yield [36]. The average milk yield per milking was highest when milking was carried out three times per day instead of once per day and when milking frequency was 3 h instead of 2 h [37]. The pH of Istrian jennies’ and Littoral Dinaric jennies’ milk (7.05, 6.86) determines was lower than the values found in previous studies [17,20,28,38,39], which ranged from 6.92 [28] to 7.49 [20].

**Figure 2:**
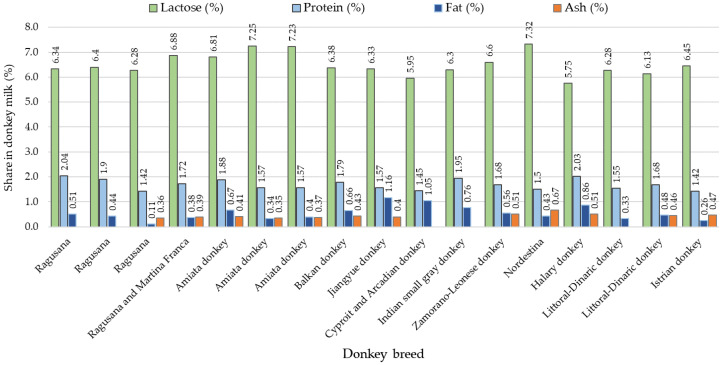
Average values of lactose, protein, fat, and ash content in milk from different donkey breeds (according to different authors) [17,18,20,21,22,23,25,26,27,28,29,38,39,40,41].

Based on the composition analysis of milk from the Istrian jennies and the Littoral Dinaric jennies, the values of lactose, protein, fat, and ash content fell within the range of the previously determined values [17,18,20,21,22,23,25,26,27,28,29,38,39,40,41]. These studies reported lactose content ranging from 5.75 to 7.25%, protein content ranging from 1.42 to 2.04, fat content ranging from 0.11 to 1.16%, and ash content ranging from 0.36 to 0.51% (Figure 2).

The count of somatic cells and microorganisms in the milk of the Istrian jennies and the Littoral Dinaric jennies was relatively low, consistent with previous research findings. Malacarne et al. [20] reported higher values of somatic cell count (SCC) and microorganism count (MC) in Ragusano jennies (4.82 log10 mL^−1^ and 4.73 log10 mL^−1^, respectively), whereas Alabiso et al. [17] and Giosuè et al. [18] found lower SCC values (3.89 log10 mL^−1^ and 3.90 log10 mL^−1^, respectively) in the same breeds. Milk from Arcadian jennies showed a low count of microorganisms and somatic cells, with an average of 4.4 log10 mL^−1^ and 4.8 log10 mL^−1^, respectively [24]. A slightly higher SCC (4.09 log10 mL^−1^) was observed in milk from Littoral Dinaric jennies in a previous study [23]. These findings indicate a high level of hygiene in donkey milk, with no significant differences observed in the counts of somatic cells and microorganisms among the studied breeds.

**Figure 3:**
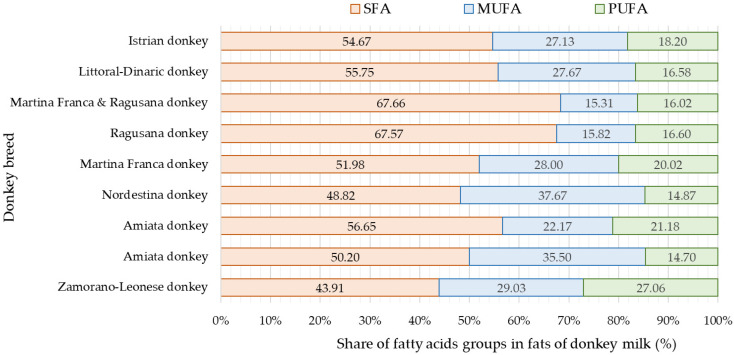
Share of saturated fatty acids (SFA), monounsaturated fatty acids (MUFA), and polyunsaturated fatty acids (PUFA) in the milk of various donkey breeds (SFA represents the sum of saturated fatty acids, MUFA represents the sum of monounsaturated fatty acids, and PUFA represents the sum of polyunsaturated fatty acids) [21,26,29,39,42,43,44].

Saturated fatty acids are more represented in the milk fat of Istrian jennies and Littoral Dinaric jennies, which aligns with findings from previous studies [21,29,42,43,44]. A higher share of UFA was detected in the milk fat of Zamorano-Leonese and Nordestina breeds [26,39] (Figure 3). The prevalence of MUFA over PUFA was consistently observed in several earlier studies [21,26,29,39,42,43]. The dominant fatty acids were two saturated fatty acids (C14:0 and C16:0) and two unsaturated fatty acids (C18:1n-9 and C18:2n-6) [38,39,42]. Notably, a significant content of linoleic fatty acids (C18:3n-3) was found in the milk of donkeys of Zamorano-Leonese and Nordestina breeds (12.32% and 6.51%, respectively) [26,39]. Some studies [26,38,42,45] reported significant levels of C8:0, C10:0, and C12:0 fatty acids, which were not observed in the milk fat of Istrian jennies and Littoral Dinaric jennies.

Indices commonly used as indicators of the health benefits of donkey milk to consumers include the UFA/SFA ratio, PUFA/SFA ratio, n-6/n-3 PUFA ratio, atherogenicity index (AI), and the thrombogenicity index (TI). The content of n-3 PUFA fatty acids in milk fat (5.50, 3.07) further confirms the favorable fatty acid profile for consumer health. Martemucci et al. [43] found lower values for the n-6/n-3 PUFA ratio, AI and TI (1.81, 1.16, and 0.70, respectively), and higher values for UFA/SFA and PUFA/SFA ratios (0.92, 0.39) in Martina Franca jennies’ milk. Cavalcanti et al. [26] observed lower index values for n-6/n-3 (1.19), AI (0.85), and TI (0.59) in Nordestina jennies’ milk. Ragona et al. [29] reported lower values for UFA/SFA, PUFA/SFA, and n-6/n-3 ratios (0.80, 0.37, 1.65) in the Amiatia donkey breed, and the same indices were lower (0.48, 0.25, 0.86) in the milk of Ragusana jennies [20]. Lazarević et al. [40] observed similar UFA/SFA, PUFA/SFA, and n 6/n-3 indices in Balkan jennies’ milk (0.81, 0.27, 1.03). The reported indices for Zamorano-Leonese jennies’ milk were 1.25, 0.62 and 1.16, respectively [39].

Donkey milk has been valued for its nutritional properties for thousands of years. Its composition, particularly in terms of protein and lipid content, closely resembles human milk, making it historically used in infant feeding. Donkey milk contains lower amounts of casein and β-lactoglobulin, which are potential allergenic components, making it a suitable alternative for individuals with a cow’s milk allergy [46,47]. The high concentration of unsaturated fatty acids in donkey milk holds importance in preventing cardiovascular, autoimmune, and inflammatory diseases [42]. Wolter [48] highlights the beneficial effects of milk on osteogenesis, atherosclerosis treatment, and the rehabilitation of patients with cardiac problems and diseases. Lysozyme, lactoperoxidase, and lactoferrin present in donkey milk are known as antimicrobial and bacteriostatic agents [39,49,50], offering potential benefits for intestinal health, particularly in individuals with weakened immune systems such as children, the elderly, and convalescents. In Croatia, donkey milk has been traditionally used for centuries to treat respiratory diseases, especially whooping cough in children, which has colloquially been referred to as “donkey cough” due to its association with the treatment. The identified indicators of local donkey breeds’ milk production potentially highlight their significant value. To fully exploit this potential, it is crucial to promote the preference for donkey milk consumption, emphasizing its potential functional and health benefits. Although donkey milk production per milking is relatively modest compared to cows, sheep, or goats, its high market price (EUR 35/kg in Croatia) has sparked interest in this type of production. It is important to note that donkey milk production is closely tied to reproduction, as new offspring are necessary for both milk and meat production. Therefore, the production of donkey milk and donkey meat should be considered in conjunction.

## 5. Conclusions

The development and improvement of models for utilizing donkey breeds in meat and/or milk production should be based on a comprehensive understanding of their production characteristics. If production indicators are solely assessed based on the quantity of meat or milk per production animal, the potential may seem modest. However, when considering the qualitative indicators of milk and meat, the production potential becomes much more promising. It is crucial to ensure that consumers are well-informed about the product itself (donkey milk and/or meat), the associated nutritional benefits, the positive impact on the community through donkey farming, the preservation of local identity, the provision of ecosystem services, and other relevant aspects. Donkey milk and/or meat production programs are interconnected, as lactation occurs after foaling, which then becomes the basis for meat production. Further research should prioritize the quality of donkey milk and meat, which hold particular interest for consumers.

## Figures and Tables

**Table 1:** Average values (LS mean ± SE and *p*-value) of the carcass characteristics for two Croatian donkey breeds.

Carcass Traits	Istrian Donkey	Littoral Dinaric Donkey	*p*-Value
Slaughter body weight (SBW), kg	177.20 ± 3.050	115.10 ± 2.354	<0.001
Cold carcass weight (CCW), kg	89.04 ± 1.363	57.00 ± 1.256	<0.001
Dressing percentage (CCW/SBW), %	50.25 ± 0.163	49.52 ± 0.113	<0.001
Muscle in rib section, %	67.10 ± 0.578	66.05 ± 0.420	0.156
Bone in rib section, %	25.96 ± 0.484	26.13 ± 0.352	0.782
Fat in rib section, %	6.94 ± 0.291	7.82 ± 0.212	0.022
Surface of LTL, cm^2^	55.63 ± 1.352	47.95 ± 1.007	<0.001
Boneless meat weight (BMW), kg	50.10 ± 1.388	30.13 ± 1.133	<0.001
Boneless meat yield (BMW/CCW), %	56.27 ± 1.338	52.86 ± 1.112	<0.001

**Table 2:** Average values (LS mean ± SE and *p*-value) of the carcass primal cuts (in kg) and the percentage of primal cuts in boneless carcasses (%) for two Croatian donkey breeds.

Primal Cuts of Donkey Meat (Quality Class)	Weight of the Primal Cuts (kg)			Share of the Primal Cuts (%)		
	ID	LDD	*p*-Value	ID	LDD	*p*-Value
Loin (E)	5.67 ± 0.43	3.71 ± 0.36	0.001	11.27 ± 0.83	12.21 ± 0.69	0.387
Neck (E)	6.68 ± 0.25	3.26 ± 0.21	<0.001	13.26 ± 0.40	10.82 ± 0.331	<0.001
Tenderloin (E)	1.84 ± 0.15	1.32 ± 0.12	0.008	3.74 ± 0.41	4.48 ± 0.34	0.169
∑ E class of meat	14.19 ± 0.67	8.29 ± 0.57	<0.001	28.32 ± 0.99	27.51 ± 0.84	0.498
Big rose (I)	3.65 ± 0.11	2.15 ± 0.09	<0.001	7.30 ± 0.14	7.17 ± 0.12	0.483
Small rose (I)	3.62 ± 0.13	2.14 ± 0.11	<0.001	7.17 ± 0.18	7.13 ± 0.15	0.854
Black fricandeau (I)	4.01 ± 0.14	2.46 ± 0.12	<0.001	8.03 ± 0.25	8.16 ± 0.21	0.690
Knuckle (I)	4.04 ± 0.18	2.53 ± 0.15	<0.001	8.08 ± 0.33	8.41 ± 0.27	0.442
∑ I class of meat	15.32 ±0.54	9.28 ± 0.46	<0.001	30.58 ± 0.46	30.80 ± 0.39	0.627
Ribs and flank (II)	6.43 ± 0.47	3.84 ± 0.39	<0.001	12.79 ± 0.95	12.46 ± 0.79	0.793
Shoulder (II)	4.78 ± 0.54	2.77 ± 0.45	0.005	9.54 ± 1.35	9.06 ± 1.12	0.784
Shank (II)	4.59 ± 0.27	2.72 ± 0.22	<0.001	9.14 ± 0.64	9.24 ± 0.53	0.904
Other cuts (II)	4.79 ± 0.34	3.23 ± 0.28	0.001	9.67 ± 0.80	10.85 ± 0.67	0.261
∑ II class of meat	20.59 ± 0.86	12.56 ± 0.74	<0.001	41.10 ± 1.20	41.69 ± 1.01	0.708

ID, Istrian donkey; LDD, Littoral Dinaric donkey; E, extra-quality class of meat: loin, neck, and tenderloin; I, first-quality class of meat: big rose, small rose, black fricandeau, and knuckle; II, E, second-quality class of meat: ribs and flank, shoulder, shank, other cuts (rest of meat).

**Table 3:** Physical characteristics and chemical composition of meat from two Croatian donkey breeds (LS Mean ± SE and *p*-value).

Meat Characteristic		Istrian Donkey	Littoral Dinaric Donkey	*p*-Value
Muscle pH at 24 h		5.50 ± 0.043	5.56 ± 0.017	0.223
Color at 24 h:	Lightness (L*)	33.15 ± 1.095	31.16 ± 0.975	0.181
	Redness (a*)	10.48 ± 0.353	11.07 ± 0.314	0.214
	Yellowness (b*)	12.22 ± 0.354	13.01 ± 0.315	0.103
	Chroma (C*)	16.17 ± 0.397	17.14 ± 0.353	0.073
	Hue (H*)	49.43 ± 1.059	49.57 ± 0.943	0.919
Moisture (%)		72.63 ± 0.220	72.10 ± 0.196	0.079
Protein (%)		23.56 ± 0.198	23.63 ± 0.176	0.787
Fat (%)		1.77 ± 0.243	2.10 ± 0.217	0.334
Ash (%)		1.13 ± 0.014	1.16 ± 0.013	0.177

**Table 4:** Average values (LS mean ± SE and *p*-value) of the fatty acid composition in the total fatty acids of the intramuscular fat in the meat of two Croatian donkey breeds (g/100 g of total fatty acids).

Fatty Acids	Istrian Donkey	Littoral Dinaric Donkey	*p*-Value
C8:0	3.42 ± 0.392	3.35 ± 0.531	0.922
C10:0	3.51 ± 0.425	3.30 ± 0.575	0.733
C12:0	1.58 ± 0.211	1.57 ± 0.286	0.970
C14:0	2.71 ± 0.220	3.20 ± 0.298	0.212
C15:0	0.34 ± 0.058	0.23 ± 0.078	0.286
C16:0	23.75 ± 0.624	24.03 ± 0.845	0.792
C16:1	4.24 ± 0.458	4.27 ± 0.621	0.972
C17:0	0.42 ± 0.051	0.38 ± 0.070	0.605
C18:0	9.38 ± 0.459	9.32 ± 0.621	0.943
C18:1n-9	24.58 ± 1.273	24.02 ± 1.723	0.799
C18:2n-6	17.19 ± 0.767	18.07 ± 0.839	0.508
C18:3n-3	2.90 ± 0.253	2.72 ± 0.342	0.682
C20:1	1.86 ± 0.220	1.74 ± 0.297	0.752
C20:4n-6	0.56 ± 0.084	0.51 ± 0.114	0.729
C20:5n-3	2.69 ± 0.150	2.50 ± 0.203	0.469
C22:5n-3	0.87 ± 0.126	0.79 ± 0.171	0.704
SFA	45.11 ± 0.806	45.38 ± 1.092	0.847
UFA	54.89 ± 0.806	54.62 ± 0.902	0.847
MUFA	30.67 ± 1.428	30.03 ± 1.933	0.792
PUFA	24.21 ± 0.978	24.59 ± 1.325	0.821
UFA/SFA	1.22 ± 0.040	1.21 ± 0.054	0.840
PUFA/SFA	0.54 ± 0.021	0.54 ± 0.029	0.869
n-6 PUFA	17.75 ± 0.780	18.57 ± 0.957	0.539
n-3 PUFA	6.47 ± 0.337	6.02 ± 0.456	0.443
n-6/n-3 PUFA	2.79 ± 0.173	3.17 ± 0.234	0.210
SCDi 16	14.78 ± 1.091	14.79 ± 1.477	0.997
SCDi 18	71.85 ± 1.906	71.80 ± 2.580	0.982
AI	0.66 ± 0.025	0.70 ± 0.033	0.298
TI	0.82 ± 0.062	0.86 ± 0.116	0.323

SFA, sum of saturated fatty acids; UFA, sum of unsaturated fatty acids; MUFA, sum of monounsaturated fatty acids; PUFA, sum of polyunsaturated fatty acids; UFA/SFA, ratio of the sum of UFA and SFA fatty acids; PUFA/SFA, ratio of the sum of PUFA and SFA fatty acids; n-6 PUFA, sum of n-6 PUFA fatty acids; n-3 PUFA, sum of n-3 PUFA fatty acids; n-6/n-3 PUFA, ratio of the sum of n-6/n-3 PUFA fatty acids; SCDi 16, stearoyl-CoA desaturase activity C16:0 and C16:1 indices; SCDi 18, stearoyl-CoA desaturase activity C18:0 and C18:1 indices; AI, atherogenic index; TI, thrombogenic index.

**Table 5:** Average values (LS mean ± SE and *p*-value) of milk yield and the characteristics of the milk for the two Croatian local donkey breeds.

Characteristic	Istrian Jennies	Littoral Dinaric Jennies	*p*-Value
Milk yield (mL/milking)	841.6 ± 31.38	442.1 ± 19.71	<0.001
pH of milk	7.05 ± 0.029	6.86 ± 0.018	<0.001
Lactose (g/100 g)	6.43 ± 0.050	6.13 ± 0.032	<0.001
Protein (g/100 g)	1.43 ± 0.060	1.67 ± 0.038	0.001
Fat (g/100 g)	0.26 ± 0.045	0.48 ± 0.028	<0.001
Ash (g/100 g)	0.47 ± 0.38	0.46 ± 0.024	0.590
SCC (log10 mL^−1^)	3.82 ± 0.055	3.85 ± 0.041	0.428
MC (log10 mL^−1^)	3.39 ± 0.074	3.40 ± 0.041	0.834

SCC, number of somatic cells in donkey milk; MC, number of microorganisms in donkey milk.

**Table 6:** Average values (LS mean ± SE and *p*-value) of the fatty acid composition in the total fatty acids of milk from Istrian jennies and Littoral Dinaric jennies (g/100 g of total fatty acids).

Fatty Acids	Istrian Jennies	Littoral Dinaric Jennies	*p*-Value
C8:0	0.87 ± 0.123	1.04 ± 0.113	0.351
C9:0	1.46 ± 0.086	2.17 ± 0.196	0.004
C10:0	3.35 ± 0.297	2.55 ± 0.255	0.058
C12:0	4.04 ± 0.280	3.36 ± 0.345	0.149
C14:0	15.91 ± 0.463	15.96 ± 0.422	0.930
C15:0	3.70 ± 0.302	3.48 ± 0.362	0.660
C16:0	21.96 ± 0.701	23.30 ± 0.515	0.130
C16:1	2.56 ± 0.284	2.89 ± 0.201	0.346
C18:0	3.43 ± 0.222	3.85 ± 0.201	0.159
C18:1n-9	24.25 ± 0.605	23.99 ± 0.633	0.780
C18:2n-6	13.78 ± 0.464	13.23 ± 0.667	0.516
C18:3n-3	4.35 ± 0.240	3.41 ± 0.243	0.013
C20:1	0.34 ± 0.064	0.77 ± 0.123	0.004
SFA	54.71 ± 0.808	55.71 ± 1.042	0.469
UFA	45.29 ± 0.808	44.29 ± 1.043	0.474
MUFA	27.15 ± 0.741	27.65 ± 0.725	0.644
PUFA	18.14 ± 0.552	16.64 ± 0.687	0.112
UFA/SFA	0.83 ± 0.028	0.80 ± 0.039	0.545
PUFA/SFA	0.33 ± 0.015	0.30 ± 0.017	0.190
n-6 PUFA	10.49 ± 1.383	13.99 ± 1.997	0.173
n-3 PUFA	5.50 ± 0.817	3.07 ± 0.358	0.014
n-6/n-3 PUFA	2.45 ± 0.449	4.97 ± 0.555	0.002
SCDi 16	10.27 ± 0.974	11.10 ± 0.783	0.525
SCDi 18	87.59 ± 0.742	86.11 ± 0.722	0.160
AI	1.99 ± 0.067	2.07 ± 0.085	0.490
TI	1.22 ± 0.081	1.52 ± 0.106	0.030

SFA, sum of saturated fatty acids; UFA, sum of unsaturated fatty acids; MUFA, sum of monounsaturated fatty acids; PUFA, sum of polyunsaturated fatty acids; UFA/SFA, ratio of the sum of UFA and SFA fatty acids; PUFA/SFA, ratio of the sum of PUFA and SFA fatty acids; n-6 PUFA, sum of n-6 PUFA fatty acids; n-3 PUFA, sum of n-3 PUFA fatty acids; n-6/n-3 PUFA, ratio of the sum of n-6/n-3 PUFA fatty acids; SCDi 16, stearoyl-CoA desaturase activity C16:0 and C16:1 indices; SCDi 18, stearoyl-CoA desaturase activity C18:0 and C18:1 indices; AI, atherogenic index; TI, thrombogenic index.

## Data Availability

The data presented in this study are available on request from the corresponding author. The data are not publicly available to preserve the privacy of the data.

## Supplementary Materials

The following supporting information can be downloaded at: https://www.mdpi.com/article/10.3390/ani13132146/s1, Figure S1: The donkey primal cuts used in this study. The lines indicate the divisions between the primal cuts; Figure S2: A dish prepared with Istrian donkey meat; Figure S3: Carpaccio made with Istrian donkey meat; Figure S4: Sausages and pate crafted using Istrian donkey meat; Figure S5: Kumis, a beverage from the milk of Istrian jennies.
