# Supplementary material for: Potential of Endangered Local Donkey Breeds in Meat and Milk Production
Source: Animals (Basel). 2023 Jun 29;13(13):2146. doi: 10.3390/ani13132146 (PMC10339952; doi:10.3390/ani13132146)
Supplement: Supplementary file 1 [file animals-13-02146-s001.zip › animals-2442160-supplementary.pdf]

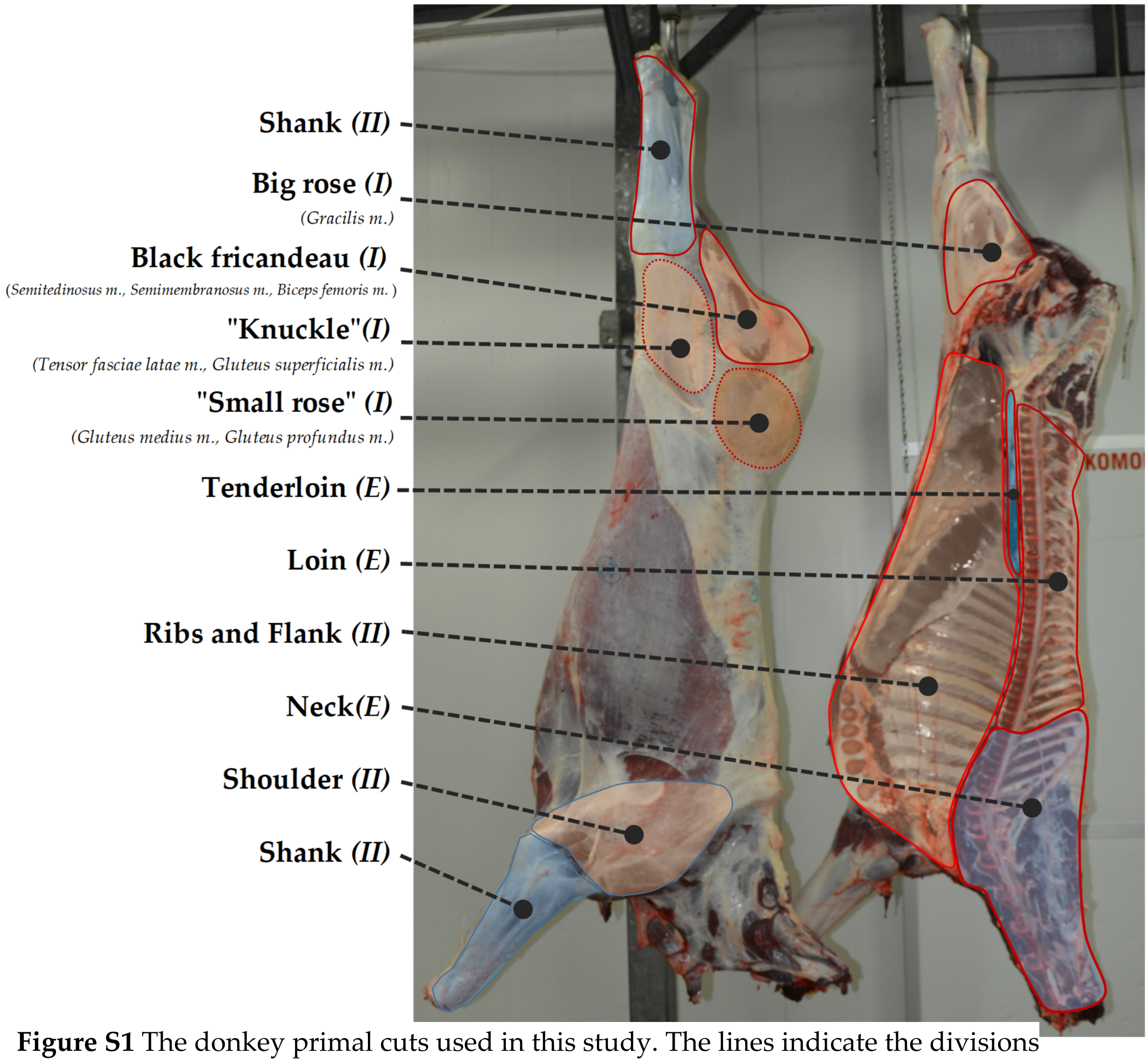

Figure S1 The donkey primal cuts used in this study. The lines indicate the divisions between the primal cuts.

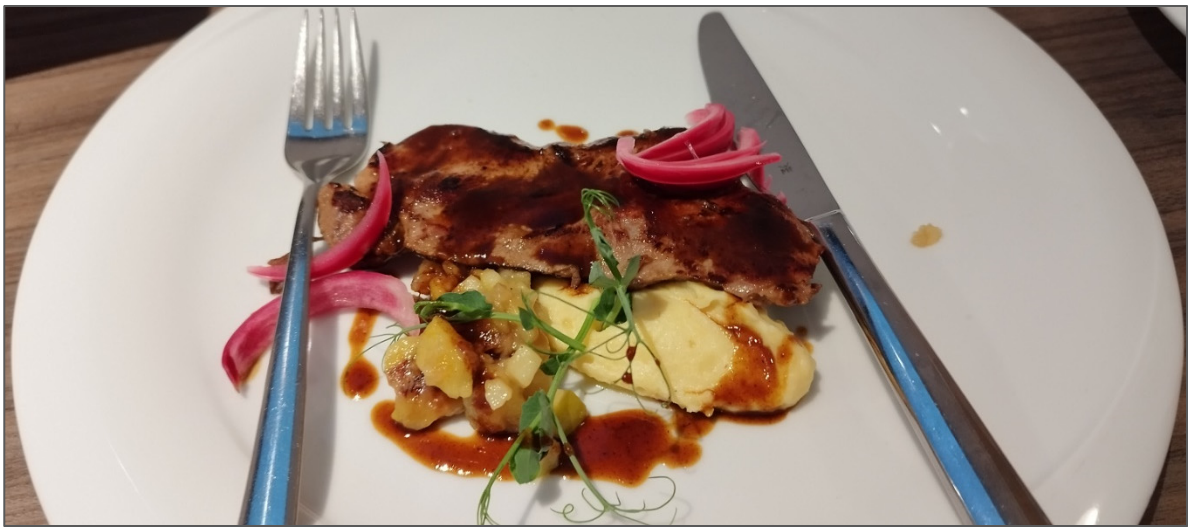

Figure S2 A dish prepared with Istrian donkey meat.

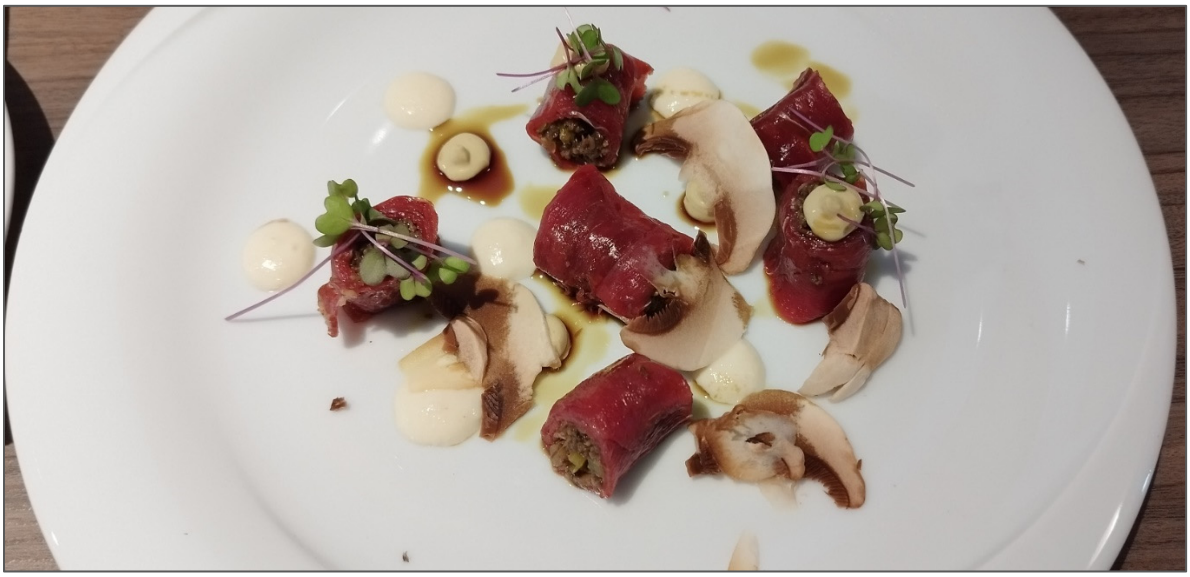

Figure S3 Carpaccio made with Istrian donkey meat.

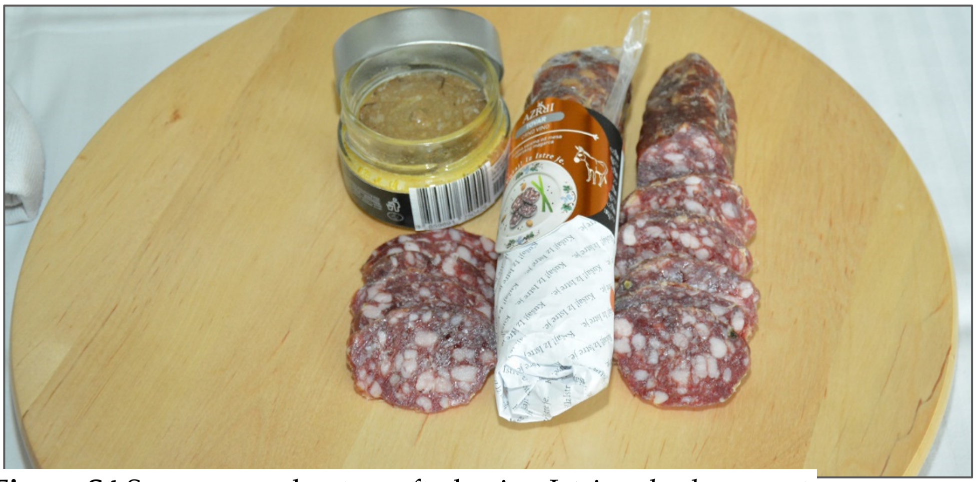

Figure S4 Sausages and pate crafted using Istrian donkey meat.

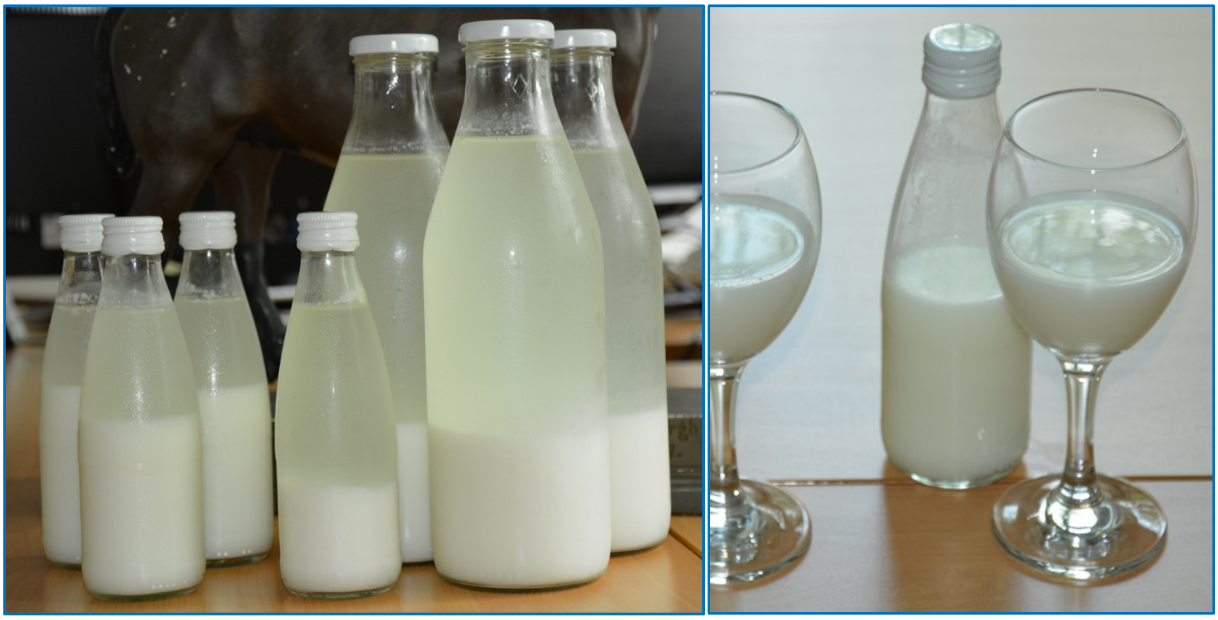

Figure S5 Kumis, a beverage from the milk of Istrian jennies.
